# Red cell distribution width and renal outcome in patients with non-dialysis-dependent chronic kidney disease

**DOI:** 10.1371/journal.pone.0198825

**Published:** 2018-06-11

**Authors:** Sayoko Yonemoto, Takayuki Hamano, Naohiko Fujii, Karin Shimada, Satoshi Yamaguchi, Ayumi Matsumoto, Keiichi Kubota, Nobuhiro Hashimoto, Tatsufumi Oka, Masamitsu Senda, Yusuke Sakaguchi, Isao Matsui, Yoshitaka Isaka

**Affiliations:** 1 Department of Nephrology, Osaka University Graduate School of Medicine, Suita, Osaka, Japan; 2 Department of Internal Medicine, Hyogo Prefectural Nishinomiya Hospital, Nishinomiya, Hyogo, Japan; 3 Department of Inter-Organ Communication Research in Kidney Disease, Osaka University Graduate School of Medicine, Suita, Osaka, Japan; The University of Tokyo, JAPAN

## Abstract

Higher red cell distribution width (RDW) has been reported to predict mortality among patients with various diseases, including chronic kidney disease (CKD). However, whether RDW is associated with renal outcome remains unclear. We investigated the relationship between RDW and renal outcome in patients with non-dialysis-dependent CKD (NDD-CKD). This prospective, observational study of patients with CKD was conducted at a single nephrology department. First, we performed regression analyses for the decline in estimated glomerular filtration rate (eGFR) during the first 3 months of observation to determine its short-term association with RDW. Next, we categorized baseline RDW into two groups by its median (13.5%) and performed Cox regression analyses to investigate whether higher RDW was an independent predictor of renal outcomes defined as a composite of the initiation of dialysis and doubling of the serum creatinine concentration. Furthermore, we repeated the analyses to confirm whether the transition of the RDW category during the first 3 months would also predict renal outcomes. We enrolled 703 patients. Baseline RDW showed a non-linear association with the eGFR decline during the first 3 months, with a greater negative correlation at the lower end of the RDW distribution. Over a median follow-up of 1.8 years, 178 patients (25.3%) reached the renal endpoint. Multivariable Cox regression analyses showed that patients with higher RDW had a higher risk of developing renal outcomes (adjusted hazard ratio [HR]: 1.47, 95% confidence interval [CI]: 1.05–2.07) than did those with lower RDW. Furthermore, patients with sustained, higher RDW demonstrated a significantly higher risk than did those with consistently lower RDW (adjusted HR: 1.65, 95% CI: 1.02–2.67). In conclusion, higher RDW was independently associated with worse renal outcome in patients with NDD-CKD. RDW could be an additional prognostic marker of the progression of CKD.

## Introduction

Red cell distribution width (RDW) is a measure of the range of variation in the red cell volume, and it is routinely reported as a part of the standard complete blood count. RDW has traditionally been used to diagnose different types of anemia, and elevated RDW is considered a marker of malnutrition (iron, vitamin B_12_, and folate deficiencies), inflammation, and other disturbances in hematopoiesis [1].

According to recent studies [2–7], RDW may be associated with mortality in various populations, including patients with kidney disease, and could be a new, independent predictor in such patients. In dialysis patients, higher RDW was associated with mortality, and was a stronger predictor of death than were traditional laboratory markers of anemia, such as transferrin saturation (TSAT) and ferritin levels [8, 9]. In a study from Taiwan on patients with chronic kidney disease (CKD) stages 3–5, higher RDW was associated with death from all-causes, cardiovascular disease (CVD), and infections [10]. However, little is known about the relationship between RDW and renal outcome, including the progression of CKD.

Several studies have already demonstrated that anemia is a strong predictor of the progression of CKD [11, 12]. Since RDW is a marker of erythropoiesis, we hypothesized that RDW would predict not only mortality, but also renal outcome. To test this hypothesis, we investigated the relationship between RDW and renal outcome in patients with non-dialysis-dependent CKD (NDD-CKD).

## Materials and methods

### Ethical considerations

The study was performed according to the ethical principles of the Declaration of Helsinki and received ethical approval for the use of an opt-out methodology by the ethics committee of Hyogo Prefectural Nishinomiya Hospital (approval number: H28-32), based on no additional burden to the patients in clinical practice.

### Study population

We enrolled consecutive outpatients with NDD-CKD who were seen between September 1, 2012 and November 30, 2016 in the nephrology department of Hyogo Prefectural Nishinomiya Hospital, Hyogo, Japan. We excluded patients who were younger than 18 years, those without biochemical data, those with an observation period that was shorter than 30 days, and those who had biopsy-proven hematologic diseases. All of the patients’ information and laboratory data were de-identified before analyses.

### Data collection and laboratory measurement

Data on baseline characteristics, including diabetes mellitus, prior CVD (history of percutaneous coronary intervention, cerebral hemorrhage, and cerebral infarction), medications (angiotensin-converting enzyme inhibitors, angiotensin II receptor blockers, erythropoiesis stimulating agents [ESAs], and iron supplements), and blood pressure (BP), were collected from patients’ medical records. The parameters of blood chemistry were measured with standard automated techniques. We calculated the estimated glomerular filtration rate (eGFR) based on inulin clearance, according to the following standard Japanese formula: 194 × creatinine^-1.094^ × age^-0.287^ (if female: × 0.739) [13]. RDW-CV, which was calculated by dividing the standard deviation (SD) of the mean cell size by the mean cell volume of red cells and multiplying by 100 to convert the value to a percentage, was routinely reported, along with the complete blood count. We used an automatic hematology analyzer (ADVIA2020, Siemens, München); the reference range for RDW-CV in our laboratory is 11.5%-14.5%. Urinary protein was measured semi-quantitatively with a dipstick test.

### Endpoint

Patients were followed until the study end date (May 31, 2017), death, or loss to follow-up. The endpoint for the study was the initiation of dialysis or doubling of the serum creatinine concentration, whichever occurred first.

### Statistical analyses

Descriptive data were summarized using proportions, means with SDs, and medians with interquartile ranges (IQRs), as appropriate. Between-group differences were analyzed using Student’s or Welch’s t test and the Mann-Whitney U test when required. Categorical variables were compared using the Chi-squared test or Fisher’s exact test when the expected numbers were small. Variables with right-skewed distribution were log-transformed before being applied to analyses. The trend analysis of RDW across CKD stages was performed by using Jonckheere-Terpstra test. Pearson’s correlation coefficients were calculated to determine the strength of associations between RDW and other laboratory parameters. For the regression analyses, we used multivariable regression, incorporating restricted cubic splines with 3 knots to investigate the potentially non-linear association between RDW and the decline in eGFR during the first 3 months of observation (defined as [eGFR at 3 months]–[eGFR at baseline]). RDW was categorized into two groups based on the median of baseline RDW (low RDW: RDW < 13.5%; high RDW: RDW ≧ 13.5%). We converted eGFR decline during the first 3 months into a binary variables that indicated negative or non-negative changes in eGFR and performed the multivariable logistic regression analysis to investigate the association between RDW and short-term eGFR decline. For survival analyses, Kaplan-Meier survival curves were generated to compare renal outcomes of patients in the two RDW categories, and the log-rank test was performed to assess the statistical significance. The relationship between RDW and renal outcomes was examined using the Cox proportional hazards model. The covariates for adjustment included (a) RDW only: unadjusted model; (b) RDW, age, sex, diabetes, prior CVD, systolic BP, and medications: adjusted model; and (c) all variables: fully adjusted model. For sensitivity analyses, we applied the Fine and Gray model to analyze the competing risk, considering death as a competing risk for the renal endpoint. Next, the propensity score (PS) of the high RDW was estimated by the logistic regression using parameters at baseline. We performed 1:1 PS matching using the greedy nearest-neighbor algorithm, with a caliper width of 0.25 of the SD of the PS’s logit and evaluated the statistical significance of the baseline RDW category as a predictor of renal outcomes. Furthermore, to evaluate the association between the transition of the RDW category and the renal endpoint, we also collected data on RDW at 3 months after the baseline. We divided patients into four groups according to the fluctuation patterns of RDW: (1) low RDW at baseline to low RDW at 3 months: L-L; (2) high-to-low: H-L; (3) low-to-high: L-H; and (4) high-to-high: H-H. We then performed Cox regression analyses to determine the composite renal outcomes. A two-tailed P-value of < 0.05 was considered significant. The statistical analyses were performed using Stata/SE 13.1 software (Stata Corp., College Station, TX).

## Results

### Patient characteristics

**Fig 1:**
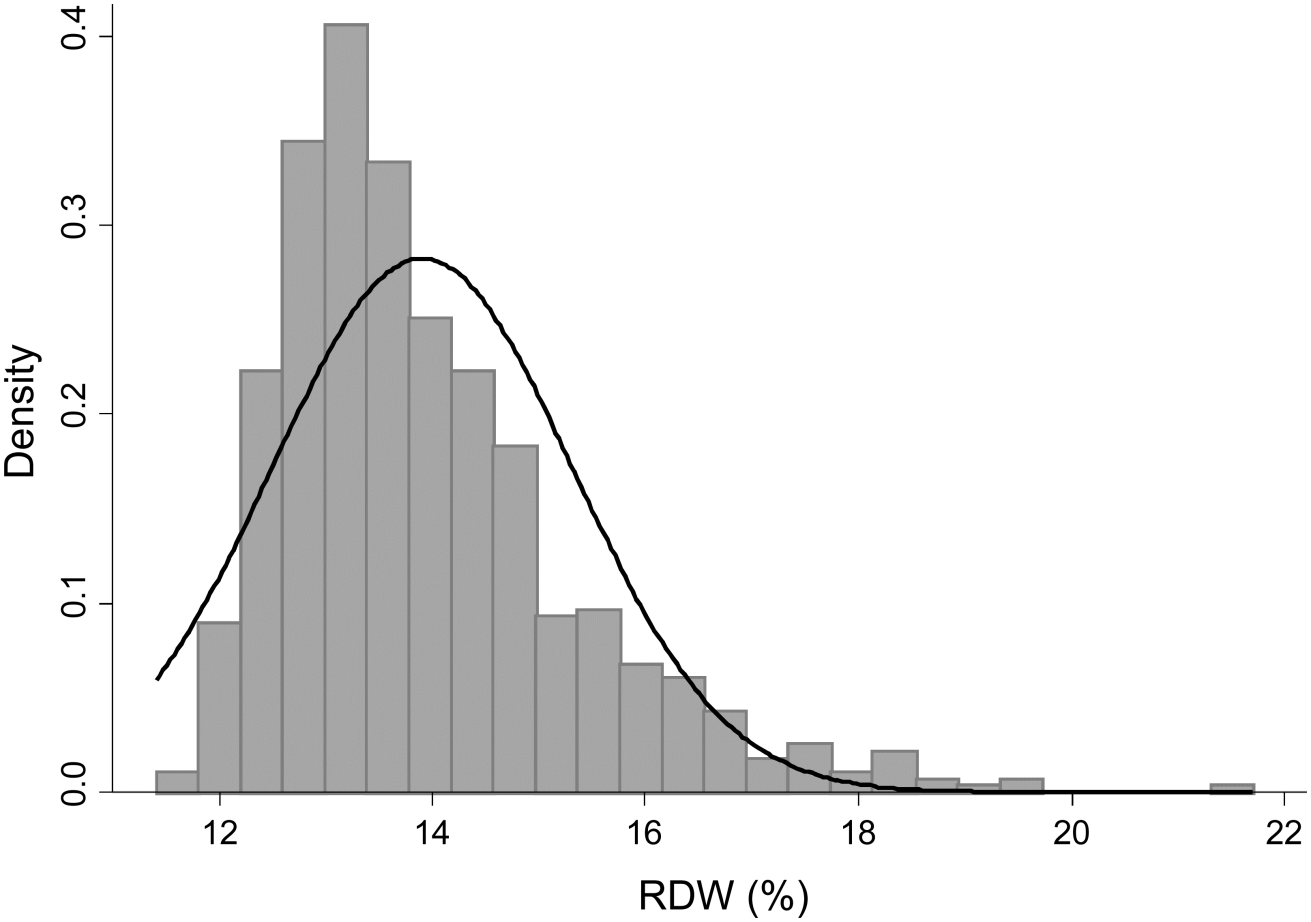
Histogram of baseline RDW. Histogram of baseline RDW of all patients (N = 703); the corresponding normal distribution is shown in the bar chart and density plot. The median RDW was 13.5%.

**Fig 2:**
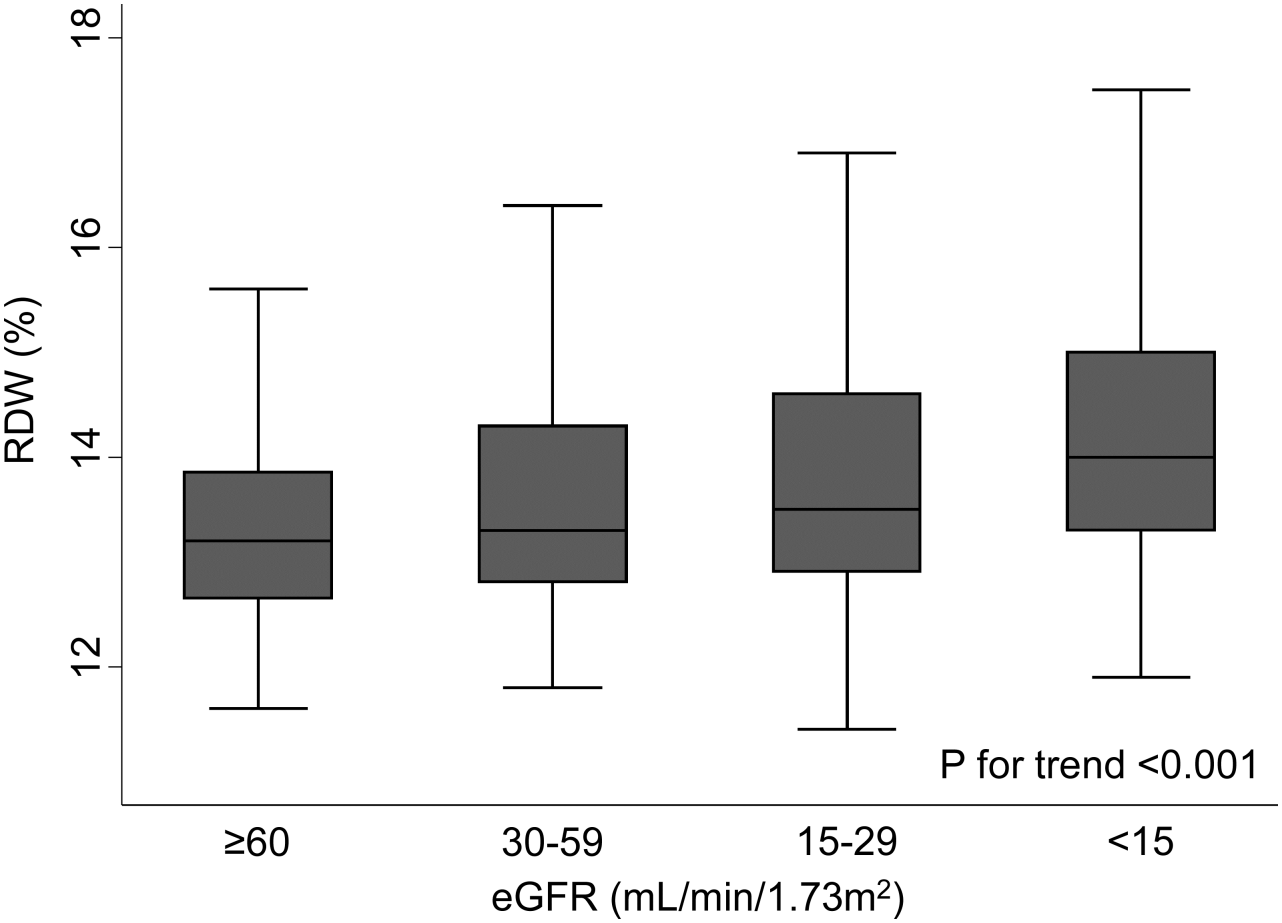
Box plots of RDW according to the eGFR categories. The box plot shows that the median RDW increased as CKD progressed. Outliers were omitted in this figure. Abbreviations: RDW, red cell distribution width; eGFR, estimated glomerular filtration rate; CKD, chronic kidney disease.

The baseline characteristics of the patients in this study are shown in Table 1. The mean age was 70.4 years (SD: 13.6 years), and about 40% of the patients were female. The proportions of patients with diabetes mellitus and prior CVD were 33.6% and 15.8%, respectively. Approximately 15% and 8% patients were treated with ESAs and iron supplements, respectively. The mean eGFR was 29.9 mL/min/1.73m^2^ (SD: 19.5 mL/min/1.73m^2^), and most patients (91.4%) had CKD stages 3, 4, or 5. The mean hemoglobin level was 11.6 g/dL (SD: 2.04 g/dL), and the median RDW was 13.5% (IQR: 12.9%-14.6%) (Fig 1). The patient characteristics across RDW categories showed that the patients in the high RDW group were more likely to be older, receive ESA therapy and iron supplements, have a history of CVD, and be proteinuric than were those in the low RDW group. They also had lower eGFR, hemoglobin, serum albumin, TSAT, and serum ferritin levels, and higher CRP levels than did those in the low RDW group. Fig 2 shows that RDW increased as the CKD stages progressed (p for trend < 0.001).

**Table 1:** Baseline characteristics categorized by the median of RDW.

Variables	All patients (N = 703)	Low RDW (<13.5) (N = 328)	High RDW (≥13.5) (N = 375)	P value
RDW (%)	13.9±1.41	12.8±0.42	14.9±1.29	<0.001
Age (years)	70.4±13.6	68.7±13.7	71.9±13.2	0.002
Gender (female)	268 (38.1%)	132 (39.1%)	136 (36.2%)	0.279
Diabetes Mellitus	236 (33.6%)	102 (31.1%)	134 (35.7%)	0.194
ESA therapy	102 (14.5%)	25 (7.6%)	77 (20.5%)	<0.001
Oral iron therapy	58 (8.3%)	17 (5.2%)	41 (10.9%)	0.006
ACE-I	53 (7.5%)	27 (8.2%)	26 (6.9%)	0.515
ARB	388 (54.9%)	180 (54.9%)	206 (54.9%)	0.988
Prior CVD	111 (15.8%)	39 (11.9%)	72 (19.2%)	0.008
sBP (mmHg)	140±22.0	138±20.1	141±23.4	0.032
dBP (mmHg)	76.2±13.4	77.2±12.5	75.3±14.1	0.051
eGFR (mL/min/1.73m^2^)	29.9±19.5	33.7±19.8	26.7±18.7	<0.001
CKD stage				
CKD stage 1&2 (eGFR ≥60)	60 (8.6%)	37 (11.3%)	23 (6.1%)	<0.001
CKD stage 3 (eGFR 30–59)	227 (32.3%)	124 (37.8%)	103 (27.5%)	
CKD stage 4 (eGFR 15–29)	247 (35.1%)	117 (35.7%)	130 (34.7%)	
CKD stage 5 (eGFR <15)	169 (24.0%)	50 (15.2%)	119 (31.7%)	
Hemoglobin (g/dL)	11.6±2.04	12.2±1.87	11.0±2.00	<0.001
MCV (fL)	93.6±6.12	93.7±4.57	93.5±7.21	0.691
Albumin (g/dL)	3.73±0.63	3.88±0.59	3.60±0.64	<0.001
CRP (mg/dL)	0.09 (0.04, 0.29)	0.07 (0.03, 0.19)	0.12 (0.05, 0.39)	<0.001
TSAT (%)	28.1±11.9	29.6±11.1	26.8±12.5	0.002
Ferritin (ng/mL)	87 (44, 158)	91 (51, 155)	83 (37, 159)	0.014
Proteinuria (≥(2+))	278 (39.6%)	101 (30.7%)	177 (47.3%)	<0.001

Values are n (%), means±SDs, and medians (1^st^ quartiles, 3^rd^ quartiles), RDW: red cell distribution width, ESA: erythropoiesis stimulating agents, ACE-I: angiotensin converting enzyme inhibitor, ARB: angiotensin II receptor blocker, CVD: cardiovascular disease, sBP: systolic blood pressure, dBP: diastolic blood pressure, eGFR: estimated glomerular filtration rate, CKD: chronic kidney disease, MCV: mean corpuscular volume, CRP: C-reactive protein, TSAT: transferrin saturation, Proteinuria: measured by the dip stick tests

### Univariate correlations between RDW and other laboratory parameters

Table 2 shows the univariate correlations between RDW and other laboratory parameters. RDW had a significant, positive correlation with age and log-transformed CRP, whereas there were negative correlations between RDW and hemoglobin, mean corpuscular volume, eGFR, serum albumin, TSAT, serum iron, log-transformed ferritin, and diastolic BP. RDW had the strongest, statistically significant, negative correlation with hemoglobin and, to the lesser extent, with eGFR, serum albumin, TSAT, and serum iron levels in patients with NDD-CKD.

**Table 2:** Univariate correlations between RDW and other laboratory parameters.

Variables	Coefficient	95% CI	P value
Age (years)	0.142	(0.068, 0.213)	<0.001
Hemoglobin (g/dL)	-0.367	(-0.430, -0.302)	<0.001
MCV (fL)	-0.092	(-0.165, -0.018)	0.015
Platelet (10^4^/μL)	0.030	(-0.044, 0.104)	0.429
eGFR (mL/min/1.73m^2^)	-0.177	(-0.248, -0.105)	<0.001
Albumin (g/dL)	-0.215	(-0.285, -0.144)	<0.001
Log CRP (mg/dL)	0.139	(0.066, 0.211)	<0.001
TSAT (%)	-0.197	(-0.267, -0.125)	<0.001
Fe (μg/dL)	-0.252	(-0.320, -0.182)	<0.001
TIBC (μg/dL)	-0.036	(-0.110, 0.038)	0.339
Log Ferritin (ng/mL)	-0.149	(-0.221, -0.076)	<0.001
sBP (mmHg)	0.067	(-0.007, 0.140)	0.077
dBP (mmHg)	-0.081	(-0.154, -0.007)	0.032

RDW: red cell distribution width, MCV: mean corpuscular volume, eGFR: estimated glomerular filtration rate, CRP: C-reactive protein, TSAT: transferrin saturation, TIBC: total iron binding capacity, sBP: systolic blood pressure, dBP: diastolic blood pressure, CI: confidence interval

### Association between RDW and short-term eGFR decline

**Fig 3:**
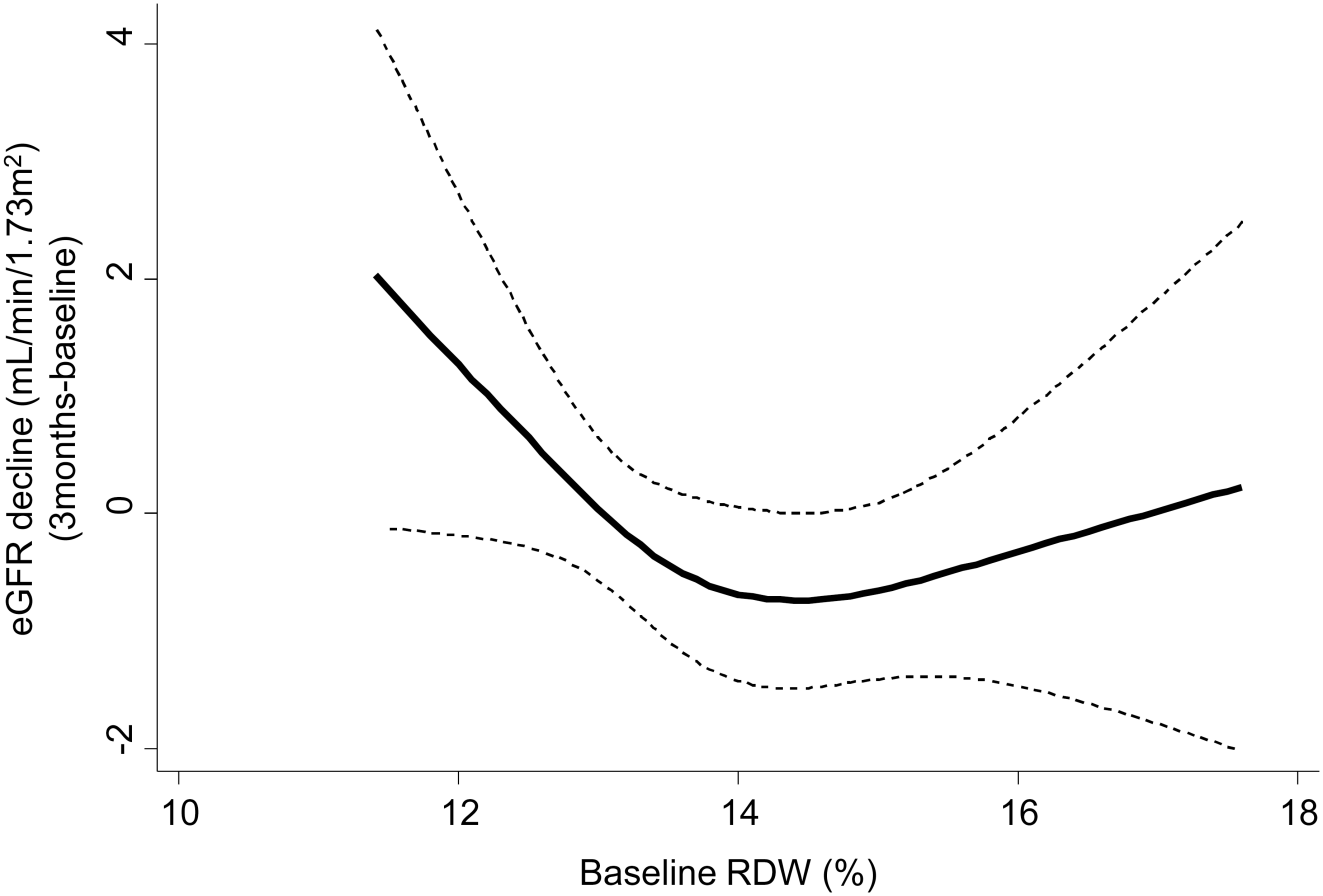
Restricted cubic spline curves for eGFR decline during the first 3 months across baseline RDW. A non-linear association between decline in the eGFR during the first 3 months and baseline RDW was observed in the multivariable regression analysis (adjusted for age, sex, diabetes mellitus, baseline eGFR, and hemoglobin, albumin, C-reactive protein, and proteinuria levels). The inflection point was located near the median of baseline RDW (13.5%). Abbreviations: RDW, red cell distribution width; eGFR, estimated glomerular filtration rate.

The eGFR decline during the first 3 months of observation had a non-linear association with baseline RDW, with a greater negative correlation in the lower half of the RDW distribution than in the greater half (Fig 3). The inflection point was located near the median of baseline RDW, which was the cut-off value of the RDW category. The multivariable logistic regression analysis showed that the patients in the high RDW group had a higher risk of worsened renal function than did those in the low RDW group (Odds Ratio: 1.49, 95% confidence interval [CI]: 1.05–2.13).

### Association between RDW and long-term renal outcome

**Fig 4:**
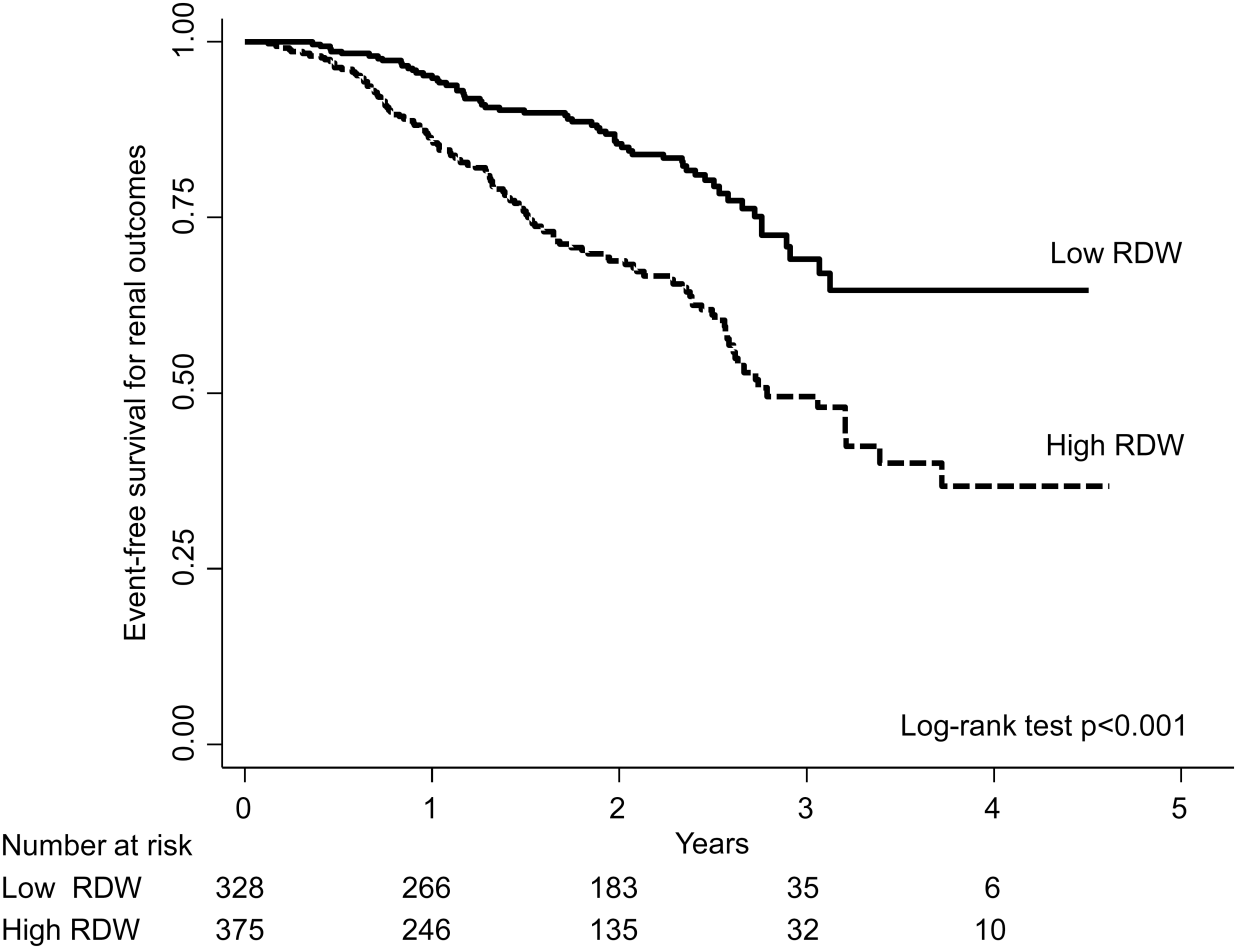
Kaplan-Meier survival curves of renal outcomes, stratified by the median of baseline RDW. RDW was categorized into two groups according to the median of baseline RDW (low RDW: < 13.5%, high RDW: ≥ 13.5%). The Kaplan-Meier survival curves of renal outcomes showed that the patients in the high RDW group had a significantly higher risk than did those in the low RDW group (p<0.001). Abbreviation: RDW, red cell distribution width.

The median follow-up period was 665 days (IQR: 345–910 days). During the observational period, 178 patients (25.3%) experienced renal outcomes (initiation of dialysis only [n = 41], doubling of the serum creatinine concentration only [n = 64], or both [n = 73]). The Kaplan-Meier survival curves of renal outcomes showed that the high RDW was associated with an increased risk compared with the low RDW (Fig 4). In the unadjusted and adjusted Cox proportional hazards models, the high RDW was consistently associated with worsened renal outcomes than did the low RDW (fully adjusted hazard ratio [HR]: 1.47, 95% CI: 1.05–2.07, Table 3). Twenty-five patients (3.6%) died without achieving the renal endpoint. Even after considering the competing risk of death, we observed a consistent, statistically significant association between the high RDW and renal outcomes (fully adjusted sub-distribution-HR: 1.47, 95% CI: 1.03–2.10). Furthermore, we performed 1:1 PS matching, in which 482 patients (S1 Table) were successfully matched. The HR of the high RDW for renal outcomes remained significant (HR: 1.68, 95% CI: 1.16–2.41).

**Table 3:** Cox regression analyses for renal outcomes stratified by the median of RDW at baseline.

	(a) Unadjusted Model		(b) Adjusted Model		(c) Fully Adjusted Model	
	HR (95% CI)	P value	HR (95% CI)	P value	HR (95% CI)	P value
High RDW	2.28 (1.67, 3.12)	<0.001	1.97 (1.42, 2.75)	<0.001	1.47 (1.05, 2.07)	0.026
Age (years)	-	-	0.99 (0.98, 1.00)	0.173	0.99 (0.98, 1.00)	0.191
Gender (Female)	-	-	0.74 (0.54, 1.03)	0.072	0.93 (0.66, 1.31)	0.671
Diabetes mellitus	-	-	2.52 (1.84, 3.44)	<0.001	1.61 (1.15, 2.27)	0.006
eGFR (ml/min/1.73m^2^)	-	-	-	-	0.95 (0.94, 0.97)	<0.001
Hemoglobin (g/dL)	-	-	-	-	0.87 (0.78, 0.96)	0.009
Albumin (g/dL)	-	-	-	-	0.73 (0.55, 0.95)	0.022
Proteinuria (≥(2+))	-	-	-	-	3.32 (2.25, 4.90)	<0.001

(a) Unadjusted Model, (b) Adjusted Model: adjusted for age, gender, comorbid conditions, (c) Fully Adjusted Model: adjusted for age, gender, comorbid conditions, hemoglobin, eGFR (estimated glomerular filtration rate), albumin, C-reactive protein, transferrin saturation, ferritin, and Proteinuria, High RDW: RDW≥13.5, Proteinuria: measured by the dip stick tests, HR: hazard ratio, CI: confidence interval

### Association between the transition of the RDW category and renal outcome

**Fig 5:**
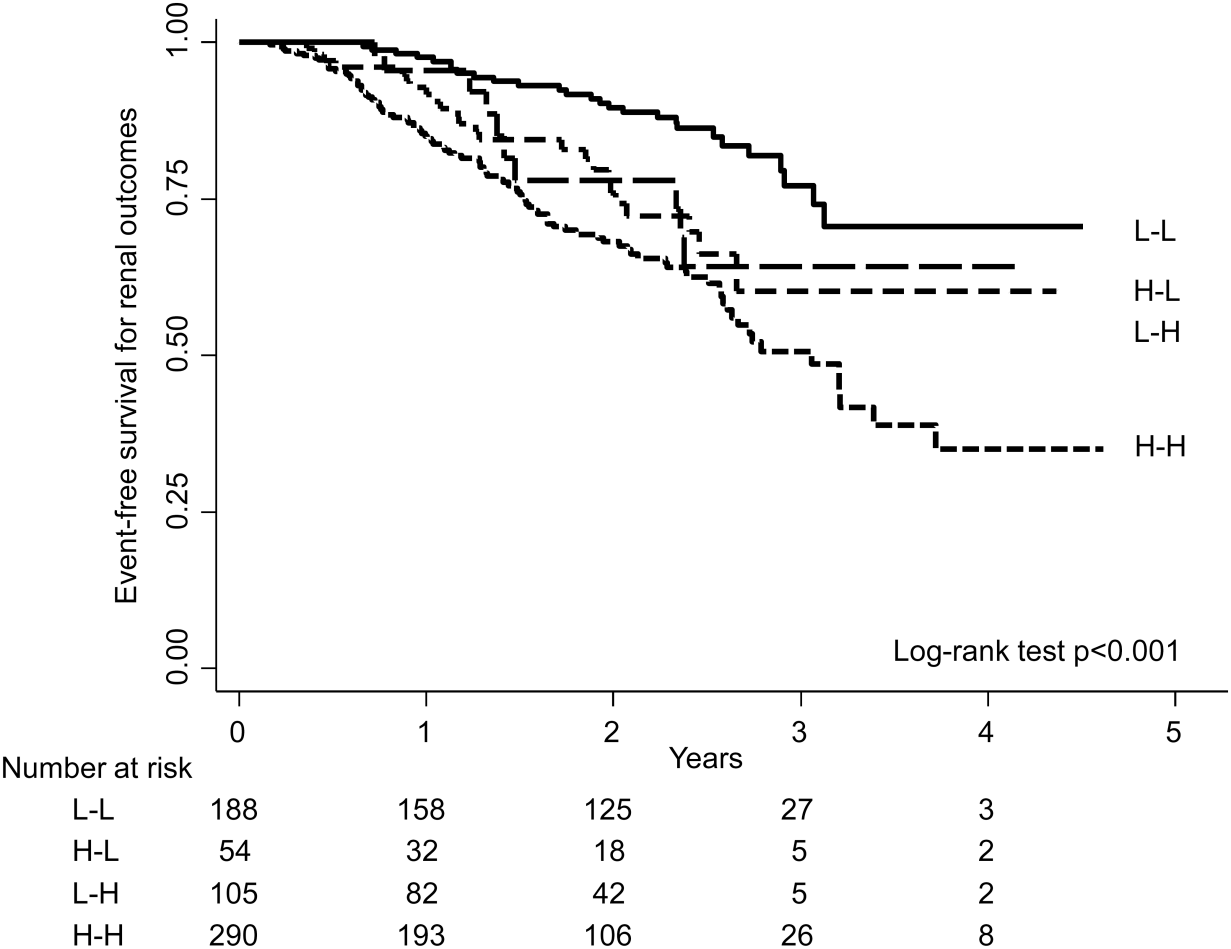
Kaplan-Meier survival curves of renal outcomes, categorized by the fluctuation patterns of RDW. We divided patients into four groups according to the fluctuation patterns of RDW during the first 3 months of observation. Patients in the H-H group showed the highest risk of having renal outcomes among these four groups (p<0.001). Abbreviations: RDW, red cell distribution width; L-L, low RDW at baseline to low RDW at 3 months; H-L, high-to-low; L-H, low-to-high; H-H, high-to-high.

We investigated the transition of the RDW category during the first 3 months of observation. About one-third of the patients were categorized into the L-L group, and 45% were categorized into the H-H group. The remaining 25% transitioned to the other RDW category at 3 months (8.5% and 16.5% in the L-H and H-L groups, respectively). As shown in Fig 5, survival curves of renal outcomes were significantly different among these groups (p<0.001), with the highest risk in the H-H group. As seen in the multivariable Cox regression analyses, patients in the H-H group showed a significantly higher risk of renal outcomes than did those in the L-L group (fully adjusted HR: 1.65, 95% CI: 1.02–2.67, Table 4).

**Table 4:** Cox regression analyses for renal outcomes stratified by the fluctuation patterns of RDW during the first three months of observation.

	(a) Unadjusted Model		(b) Adjusted Model		(c) Fully Adjusted Model	
	HR (95% CI)	P value	HR (95% CI)	P value	HR (95% CI)	P value
L-L	Ref.	-	Ref.	-	Ref.	-
H-L	1.97 (0.95, 4.08)	0.068	1.83 (0.86, 3.90)	0.116	1.26 (0.58, 2.77)	0.557
L-H	2.19 (1.26, 3.80)	0.006	2.10 (1.20, 3.67)	0.009	1.50 (0.83, 2.72)	0.177
H-H	3.21 (2.09, 4.92)	<0.001	2.86 (1.83, 4.46)	<0.001	1.65 (1.02, 2.67)	0.040

(a) Unadjusted Model, (b) Adjusted Model: adjusted for age, gender, comorbid conditions, and medications, (c) Fully Adjusted Model: adjusted for age, gender, comorbid conditions, medications, hemoglobin, estimated glomerular filtration rate, albumin, C-reactive protein, transferrin saturation, ferritin, and proteinuria, RDW: red cell distribution width, L-L: low RDW at baseline to low RDW at 3 months, H-L: high-to-low, L-H: low-to-high, H-H: high-to-high, HR: hazard ratio, CI: confidence interval

## Discussion

In this study, we demonstrated that higher RDW was independently associated with the decline in renal function in the short-term observational period. In addition, patients with NDD-CKD and higher RDW showed poor renal outcomes, which was defined as a composite of the initiation of dialysis and doubling of the serum creatinine concentration. We also revealed that sustained, higher RDW within the first 3 months of observation was a significant risk factor of poor renal outcomes in this population.

In 2007, Felker et al. first reported that RDW was associated with mortality in patients with congestive heart failure and cardiovascular disease [2]. Subsequently, various studies confirmed that higher RDW was associated with higher mortality in patients with stroke, peripheral artery disease, and acute kidney injury. Interestingly, this relationship was also observed in the general elderly population [3–6]. Very recently, Vashistha et al. demonstrated that there was a significant association between higher RDW and mortality in a large-cohort study of hemodialysis patients and that RDW had a greater prognostic value for death than did other conventional markers of anemia, such as hemoglobin, TSAT, and serum ferritin levels [8]. Similar results were obtained in patients with peritoneal dialysis [9], patients with NDD-CKD [10], and kidney transplant recipients [14]. Thus, RDW is a reliable risk factor with regard to mortality at any stage of CKD.

As for the relationship between RDW and renal function, Lippi et al. reported that there was an inverse, step-wise association between RDW and kidney function in a large cross-sectional study of 8,585 adult outpatients [15]. A similar result was observed in kidney transplant recipients [16]. Another cross-sectional study revealed that elevated RDW was independently associated with a higher risk of microalbuminuria, which is a marker of renal injury [17]. However, because of the cross-sectional nature of their study design, the association between RDW and the progression of CKD remained unclear.

In this longitudinal study, we revealed, for the first time, that there was a significant association between higher RDW and poor renal outcome in patients with NDD-CKD. Although we could not determine plausible mechanisms that would explain this association, we have some speculations regarding this relationship. First, anemia is a possible cause of the ability of RDW to predict renal outcome. Anemia was reported to be an important predictor of renal outcome [11, 12], and correction of anemia prevented the progression of CKD [18, 19]. High RDW may indicate alteration of the lifespan of erythrocytes, which usually occurs with the lack of serum erythropoietin levels during the progression of CKD. In addition, iron deficiency and inflammation also play important roles in elevation of RDW in patients with NDD-CKD [20]. In the present study, however, the association between RDW and the progression of CKD was independent of hemoglobin levels, which was considered to be a mediator in those ineffective erythropoiesis, iron deficiency, and inflammatory axes. Second, endothelial dysfunction may be another mechanism that accounts for our observations. Solak et al. demonstrated that RDW was independently related to endothelial dysfunction, as assessed with flow-mediated dilatation and the thickness of the carotid intima in patients with CKD [21]. Endothelial dysfunction might result in renal vascular damage and dysfunction. Finally, oxidative stress is a potential underlying biological mechanism that elevates RDW in the progression of CKD. Previous studies have shown that oxidative stress increased in patients with CKD compared with healthy subjects [22], and that blood selenium, which is known as one of antioxidants in humans, decreased as CKD progressed [23]. Interestingly, in humans with selenium deficiency, selenium supplementation increased glutathione peroxidase activity in erythrocytes [24, 25], thereby improving red cell survival. The interaction between oxidative stress and the erythroid maturation [26–28] might lead to an increase in RDW in patients with CKD.

In the present study, not only higher RDW at baseline but also sustained, higher RDW during the first 3 months of observation was a significant risk factor of poor renal outcome. RDW may decrease in response to therapeutic interventions, such as correction of anemia (using ESAs, iron supplements, or other nutritional supplements) and the improvement of underlying diseases (e.g., infection or malignancy). Sustained, higher RDW indicated that there were factors that could not be corrected, or that the patient’s problems remained unchanged, which might explain our result that HRs for renal outcomes in the patients with sustained, higher RDW were higher than in the patients with higher RDW only at baseline.

Our study has several limitations. First, given the observational nature of the study, we could not establish that there was a cause-and-effect relationship between RDW and renal outcome. Second, even with thorough adjustments for potential confounders, we might have missed possible important confounders, such as vitamin B12 and folate. Third, since this study was conducted at a single center, our results must be confirmed in other settings before they are generalized externally. Despite these limitations, our study may have a large impact on patients with CKD by discriminating high-risk patients.

In conclusion, we demonstrated that there was a significant association between high RDW and poor renal outcome. Although the pathophysiologic link was not explored in the present study, integrated interactions among anemia, nutritional state, inflammation, endothelial dysfunction, and oxidative stress may explain the observed association. RDW can be measured in daily clinical practice at no additional cost, therefore, this variable could be a convenient method to identify patients at a high risk of having poor renal outcome.

## Supporting information

S1 Table Baseline characteristics categorized by the median of RDW (PS matching cohort). (DOCX)

## References

[1] Evans TC, Jehle D. The red blood cell distribution width. J Emerg Med. 1991; 9 Suppl 1: 71–4. 1955687 10.1016/0736-4679(91)90592-4

[2] Felker GM, Allen LA, Pocock SJ, Shaw LK, McMurray JJ, Pfeffer MA, et al Red cell distribution width as a novel prognostic marker in heart failure: data from the CHARM Program and the Duke Databank. J Am Coll Cardiol. 2007; 50: 40–7 doi: 10.1016/j.jacc.2007.02.067 17601544 10.1016/j.jacc.2007.02.067

[3] Ani C, Ovbiagele B. Elevated red blood cell distribution width predicts mortality in persons with known stroke. J Neurol Sci. 2009; 277: 103–8. doi: 10.1016/j.jns.2008.10.024 19028393 10.1016/j.jns.2008.10.024

[4] Patel KV, Semba RD, Ferrucci L, Ferrucci L, Newman AB, Fried LP, et al Red cell distribution width and mortality in older adults: a meta-analysis. J Gerontol A Biol Sci Med Sci. 2010; 65: 258–65. doi: 10.1093/gerona/glp163 19880817 10.1093/gerona/glp163 PMC2822283

[5] Ye Z, Smith C, Kullo IJ. Usefulness of red cell distribution width to predict mortality in patients with peripheral artery disease. Am J Cardiol. 2011; 107: 1241–5. doi: 10.1016/j.amjcard.2010.12.023 21296321 10.1016/j.amjcard.2010.12.023 PMC3209662

[6] Oh HJ, Park JT, Kim JK, Yoo DE, Kim SJ, Han SH, et al Red blood cell distribution width is an independent predictor of mortality in acute kidney injury patients treated with continuous renal replacement therapy. Nephrol Dial Transplant. 2012; 27: 589–94. doi: 10.1093/ndt/gfr307 21712489 10.1093/ndt/gfr307

[7] Luo R, Hu J, Jiang L, Zhang M. Prognostic Value of Red Blood Cell Distribution Width in Non-Cardiovascular Critically or Acutely Patients: A Systematic Review. PLoS ONE. 2016;11(12): e0167000 doi: 10.1371/journal.pone.0167000 27936006 10.1371/journal.pone.0167000 PMC5147853

[8] Vashistha T, Streja E, Molnar MZ, Rhee CM, Moradi H, Soohoo M, et al Red Cell Distribution Width and Mortality in Hemodialysis Patients. Am J Kidney Dis. 2016; 68:110–21. doi: 10.1053/j.ajkd.2015.11.020 26786297 10.1053/j.ajkd.2015.11.020 PMC4921311

[9] Hsieh YP, Tsai SM, Chang CC, Kor CT, Lin CC. Association between red cell distribution width and mortality in patients undergoing continuous ambulatory peritoneal dialysis. Sci Rep. 2017; 7:45632 doi: 10.1038/srep45632 28367961 10.1038/srep45632 PMC5377316

[10] Hsieh YP, Chang CC, Kor CT, Yang Y, Wen YK, Chiu PF. The Predictive Role of Red Cell Distribution Width in Mortality among Chronic Kidney Disease Patients. PLoS ONE. 2016; 11(12): e0162025 doi: 10.1371/journal.pone.0162025 27906969 10.1371/journal.pone.0162025 PMC5132319

[11] De Nicola L, Provenzano M, Chiodini P, Borrelli S, Garofalo C, Pacilio M, et al Independent Role of Underlying Kidney Disease on Renal Prognosis of Patients with Chronic Kidney Disease under Nephrology Care. PLoS ONE. 2015; 10(5): e0127071 doi: 10.1371/journal.pone.0127071 25992629 10.1371/journal.pone.0127071 PMC4439030

[12] Inaguma D, Imai E, Takeuchi A, Ohashi Y, Watanabe T, Nitta K, et al Chronic Kidney Disease Japan Cohort Study Group: Risk factors for CKD progression in Japanese patients: findings from the Chronic Kidney Disease Japan Cohort (CKD-JAC) study. Clin Exp Nephrol. 2017; 21:446–456. doi: 10.1007/s10157-016-1309-1 27412450 10.1007/s10157-016-1309-1 PMC5486452

[13] Matsuo S, Imai E, Horio M, Yasuda Y, Tomita K, Nitta K, et al Collaborators developing the Japanese equation for estimated GFR: Revised equations for estimated GFR from serum creatinine in Japan. Am J Kidney Dis. 2009; 53: 982–92. doi: 10.1053/j.ajkd.2008.12.034 19339088 10.1053/j.ajkd.2008.12.034

[14] Mucsi I, Ujszaszi A, Czira ME, Novak M, Molnar MZ. Red cell distribution width is associated with mortality in kidney transplant recipients. Int Urol Nephrol. 2014; 46: 641–51. doi: 10.1007/s11255-013-0530-z 23959402 10.1007/s11255-013-0530-z

[15] Lippi G, Targher G, Montagnana M, Salvaqno GL, Zoppini G, Guidi GC. Relationship between red blood cell distribution width and kidney function tests in a large cohort of unselected outpatients. Scand J Clin Lab Invest. 2008; 68: 745–8. doi: 10.1080/00365510802213550 18618369 10.1080/00365510802213550

[16] Ujszaszi A, Molnar MZ, Czira ME, Novak M, Mucsi I. Renal function is independently associated with red cell distribution width in kidney transplant recipients: a potential new auxiliary parameter for the clinical evaluation of patients with chronic kidney disesase. Br J Haematol. 2013; 161: 715–25. doi: 10.1111/bjh.12315 23530521 10.1111/bjh.12315

[17] Afonso L, Zalawadiya SK, Veeranna V, Panaich SS, Niraj A, Jacob S. Relationship between red cell distribution width and microalbuminuria: a population-based study of multiethnic representative US adults. Nephron Clin Pract. 2011; 119(4): c277–82. doi: 10.1159/000328918 21921640 10.1159/000328918

[18] Rossert J, Levin A, Roger SD, Hoerl WH, Fouqueray B, Gassmann-Mayer C, et al Effect of early correction of anemia on the progression of CKD. Am J Kidney Dis. 2006; 47: 738–50. doi: 10.1053/j.ajkd.2006.02.170 16632012 10.1053/j.ajkd.2006.02.170

[19] Tsubakihara Y, Gejyo F, Nishi S, Iino Y, Watanabe Y, Suzuki M, et al High target hemoglobin with erythropoiesis-stimulating agents has advantages in the renal function of non-dialysis chronic kidney disease patients. Ther Apher Dial. 2012; 16: 529–40. doi: 10.1111/j.1744-9987.2012.01082.x 23190512 10.1111/j.1744-9987.2012.01082.x

[20] Lippi G, Targher G, Montagnana M, Salvaqno GL, Zoppini G, Guidi GC. Relation between red blood cell distribution width and inflammatory biomarkers in a large cohort of unselected outpatients. Aech Pathol Lab Med. 2009; 133: 628–32. 10.5858/133.4.628 19391664

[21] Solak Y, Yilmaz MI, Saglam M, Caqlar K, Verim S, Unal HU, et al Red cell distribution width is independently related to endothelial dysfunction in patients with chronic kidney disease. Am J Med Sci. 2014; 347: 118–24. doi: 10.1097/MAJ.0b013e3182996a96 23928544 10.1097/MAJ.0b013e3182996a96

[22] Oberg BP, McMenamin E, Lucas FL, McMonagle E, Morrow J, Ikizler TA, et al Increased prevalence of oxidant stress and inflammation in patients with moderate to severe chronic kidney disease. Kidney Int. 2004; 65: 1009–16. doi: 10.1111/j.1523-1755.2004.00465.x 14871421 10.1111/j.1523-1755.2004.00465.x

[23] Zachara BA, Salak A, Koterska D, Manitius J, Wasowicz W. Selenium and glutathione peroxidases in blood of patients with different stages of chronic renal failure. J Trace Elem Med Biol. 2004; 17: 291–9. doi: 10.1016/S0946-672X(04)80031-2 15139391 10.1016/S0946-672X(04)80031-2

[24] Alfthan G, Xu GL, Tan WH, Aro A, Wu J, Yang YX, et al Selenium supplementation of children in a selenium-deficient area in China. Biol Trace Elem Res. 2000; 73:113–125. doi: 10.1385/BTER:73:2:113 11049204 10.1385/BTER:73:2:113

[25] Thomson CD, Robinson MF, Butler JA, Whanger PD. Long-term supplementation with selenite and selenomethionine: selenium and glutathione peroxidase (EC 1.11.19) in blood components of New Zealand women. Br J Nutr. 1993; 69:577–588. 8490010 10.1079/bjn19930057

[26] Friedman JS, Lopez MF, Fleming MD, Rivera A, Martin FM, Welsh ML, et al SOD2-deficiency anemia: protein oxidation and altered protein expression reveal targets of damage, stress response, and antioxidant responsiveness. Blood. 2004; 104: 2565–73. doi: 10.1182/blood-2003-11-3858 15205258 10.1182/blood-2003-11-3858

[27] Mohanty JG, Nagababu E, Friedman JS, Rifkind JM. SOD2 deficiency in hematopoietic cells in mice results in reduced red blood cell deformability and increased heme degradation. Exp Hematol. 2013; 41: 316–21. doi: 10.1016/j.exphem.2012.10.017 23142655 10.1016/j.exphem.2012.10.017 PMC3741644

[28] Semba RD, Patel KV, Ferrucci L, Sun K, Roy CN, Guralnik JM, et al Serum antioxidants and inflammation predict red cell distribution width in older women: the Women’s Health and Aging Study I. Clin Nutr. 2010; 29: 600–4. doi: 10.1016/j.clnu.2010.03.001 20334961 10.1016/j.clnu.2010.03.001 PMC3243048

