# Supplementary material for: Red cell distribution width and renal outcome in patients with non-dialysis-dependent chronic kidney disease
Source: PLoS One. 2018 Jun 11;13(6):e0198825. doi: 10.1371/journal.pone.0198825 (PMC5995355; doi:10.1371/journal.pone.0198825)
Supplement: S1 Table — (DOCX) [file pone.0198825.s001.docx]

**S1 Table. Baseline characteristics categorized by the median of RDW (PS matching cohort)**

| Variables | All patients  (N=482) | Low RDW (<13.5)  (N=241) | High RDW (≥13.5)  (N=241) | P value |
| --- | --- | --- | --- | --- |
| RDW (%) | 13.8±1.22 | 12.9±0.41 | 14.7±1.08 | <0.001 |
| Age (years) | 70.4±13.8 | 69.8±13.7 | 71.0±14.0 | 0.312 |
| Gender (female) | 185 (38.4%) | 94 (39.0%) | 91 (37.8%) | 0.779 |
| Diabetes Mellitus | 166 (34.4%) | 80 (33.2%) | 86 (35.7%) | 0.565 |
| ESA therapy | 53 (11.0%) | 24 (10.0%) | 29 (12.0%) | 0.467 |
| Oral iron therapy | 32 (6.6%) | 14 (5.8%) | 18 (7.5%) | 0.464 |
| ACE-I | 39 (8.1%) | 22 (9.1%) | 17 (7.1%) | 0.404 |
| ARB | 261 (54.1%) | 132 (54.8%) | 129 (53.5%) | 0.784 |
| Prior CVD | 72 (14.9%) | 33 (13.7%) | 39 (16.2%) | 0.443 |
| sBP (mmHg) | 140±20.9 | 139±19.6 | 140±22.1 | 0.755 |
| dBP (mmHg) | 76.4±12.9 | 77.3±12.8 | 75.5±13.0 | 0.138 |
| eGFR (mL/min/1.73m^2^) | 30.4±19.3 | 30.9±19.0 | 29.9±19.7 | 0.587 |
| CKD stage |  |  |  |  |
| CKD stage 1&2 (eGFR ≥60) | 37 (7.7%) | 18 (7.5%) | 19 (7.9%) | 0.500 |
| CKD stage 3 (eGFR 30-59) | 166 (34.4%) | 84 (34.9%) | 82 (33.2%) |  |
| CKD stage 4 (eGFR 15-29) | 176 (36.5%) | 95 (39.4%) | 81 (32.0%) |  |
| CKD stage 5 (eGFR <15) | 103 (21.4%) | 44 (18.3%) | 59 (27.5%) |  |
| Hemoglobin (g/dL) | 11.7±1.88 | 11.8±1.77 | 11.6±1.98 | 0.187 |
| MCV (fL) | 93.7±5.73 | 93.8±4.51 | 93.7±6.75 | 0.814 |
| Albumin (g/dL) | 3.73±0.63 | 3.76±0.63 | 3.71±0.62 | 0.338 |
| CRP (mg/dL) | 0.09 (0.04, 0.27) | 0.08 (0.04, 0.26) | 0.09 (0.04, 0.27) | 0.756 |
| TSAT (%) | 28.2±11.1 | 28.6±10.6 | 27.9±11.7 | 0.534 |
| Ferritin (ng/mL) | 86 (45, 157) | 82 (48, 150) | 86 (41, 163) | 0.508 |
| Proteinuria (≥(2+)) | 186 (38.6%) | 89 (36.9%) | 97 (40.2%) | 0.454 |

RDW: red cell distribution width, ESA: erythropoiesis stimulating agents, ACE-I: angiotensin converting enzyme inhibitor, ARB: angiotensin II receptor blocker, CVD: cardiovascular disease, sBP: systolic blood pressure, dBP: diastolic blood pressure, eGFR: estimated glomerular filtration rate, CKD: chronic kidney disease, MCV: mean corpuscular volume, CRP: C-reactive protein, TSAT: transferrin saturation, Proteinuria: measured by the dip stick tests
